# Celebrating 21 years and introducing the 21st anniversary issue

**DOI:** 10.4102/sajhivmed.v22i1.1317

**Published:** 2021-10-15

**Authors:** Yunus Moosa, Lauren Jankelowitz

**Affiliations:** 1 Department of Medicine, Faculty of Internal Medicine, University of KwaZulu-Natal, Durban, South Africa, South Africa; 2 Southern African HIV Clinicians Society, Johannesburg, South Africa

The *Southern African Journal of HIV Medicine* (SAJHIVMED) is focused on HIV, in particular disease prevention and treatment, and includes topics relevant to clinical and public health practices. The primary purpose of the SAJHIVMED is to disseminate original research; however, the journal also includes editorials, case reports and series, clinical reviews, evidence based clinical practice guidelines, national guidelines, and other correspondence relevant to the field of HIV.

It has been 21 years since the inaugural publication of the SAJHIVMED in June 2000. Up until 2016, the journal was published quarterly. Since then, articles have been published online as they become available, with all the articles compiled into a single printed copy at the end of the year. Supplements have been published intermittently to address special themes or mark events, such as the Southern African HIV Clinicians Society’s (SAHCS’) biennial conference. Since 2020 and the onset of the COVID-19 pandemic, annual editions have been published as online e-books only.

During the past 21 years, over 660 articles, 50 guidelines and 45 case reports have been published in the journal, covering diverse areas ranging from basic science to clinical practice within the field of HIV, as well as areas closely related to the field. Authors and contributors range from local to the Southern African region, to global experts, providing the journal with a vast breadth of varied inputs.

The SAJHIVMED, which already boasted Scientific Electronic Library Online South Africa (SciELO SA); SCOPUS; Clarivate Analytics Web of Science Core Collection, Science Citation Index Expanded (SCIE/ISI [Institute for Scientific Information]); African Index Medicus; African Journals Online; Directory of Open Access Journals; Ebscohost; Embase; Gale, Cengage Learning; Google Scholar; and ProQuest indexing, and was added to the Norwegian Register for Scientific Journals, Series and Publishers, Level 1, in 2017, was awarded PubMed Central accreditation status in 2018.

By being an open access journal, all content is freely available at no cost to the reader or institution. In accordance with international best practice and in alignment with the Budapest Open Access Initiative (BOAI; https://www.budapestopenaccessinitiative.org/), users are allowed to read, download, copy, distribute, print, search or link to the full texts of the articles, or use them for any other lawful purpose, without asking prior permission from the publisher or the author.

Following confirmation of all relevant documentation, such as ethics clearance, the manuscript is then reviewed initially by the Editor-in-Chief with a view to establishing appropriateness of scope for the journal. Following this the blinded manuscript is assigned to at least 2 independent external reviewers. Reviewer feedback is then sent to the authors and a process of finalisation begins between the author/s and the Editors.

At the time of the launch of the journal in 2000, Prof. Des Martin (MBBCH, MMed, FCPath, DTM&H, DPH), the first President of the SAHCS and the first Editor-in-Chief of the SAJHIVMED wrote:

This first issue of the Southern African Journal of HIV Medicine coincides with the International AIDS conference in Durban, SA, and it is hoped that it will find a place in the reading of medical, scientific and allied professionals dealing with HIV disease in our region. It is also hoped that it will receive broader recognition amongst the international community, which is urged to engage actively in the discourse surrounding the epidemic in our region.^1^

He went on to say:

The Journal will provide a home for original scientific articles, review articles and continuing medical education and will also provide a forum for debate and discussion on the topical issues of the day.^1^

**FIGURE 1:**
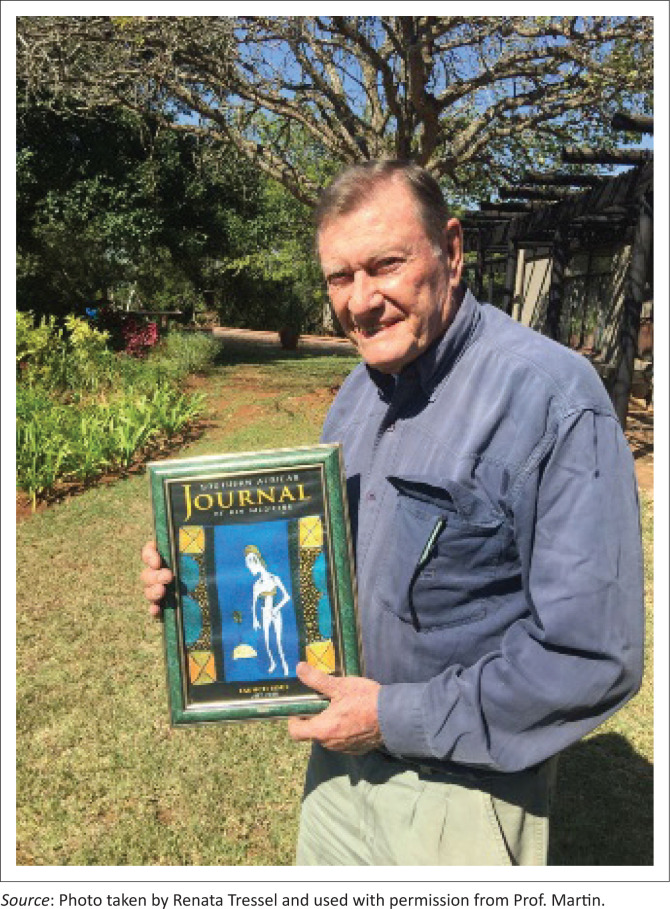
Prof. Des Martin, Founding Editor of the Southern African Journal of HIV Medicine, May 2021, with his framed copy of the first edition.

Prof. Des Martin (Figure 1), now retired to Mpumalanga, was involved in the field of HIV medicine at various levels for over 35 years. Martin held a number of prestigious positions, including Past President of SAHCS from 1998 to 2008; Editor-in-Chief of the SAJHIVMED; Deputy Director of the National Institute for Virology; Convenor of Examiners for the Colleges of Medicine of South Africa; Chair of the Basic Sciences Track at the 13th International Aids Conference, Durban, SA, in 2000; visiting Professor Johns Hopkins Hospital Baltimore (1998); and Professor in the Department of Clinical Virology, Faculty of Health Sciences, University of Pretoria.

In May 2021, Prof. Martin (personal communication) commented that:

‘The Southern African Journal of HIV Medicine was born in July 2000, as it was launched at the IAS Durban 2000 International AIDS conference. These were particularly vexed times in the local HIV/AIDS arena because of the intense activity of the “AIDS dissidents” who had found favour with a number of prominent persons in South Africa and beyond. The “Durban Declaration” was formulated by a number of the foremost HIV scientists of the day, including many from the Society, which effectively countered the misinformation spread by the dissidents. The Journal has now “come of age” and continues to provide education, teaching, dissemination of the results of the latest local research and much more to the HIV-community of our region, and indeed, of Africa. Congratulations to all who contribute to the publication of the Journal.’

There have been a further five Editors-in-Chief, all of whom have contributed immensely to the growth and credibility of the SAJHIVMED. Dr Shaun Conway, who co-created the Journal, spent a brief time as its first Managing Editor whilst serving as the Director of SAHCS. He was also the founder of Right-to-Care, co-founder of Re-Action! and founded numerous digital innovation start-ups. He worked as an advisor to the World Health Organization (WHO), and as the HIV and Health Systems Advisor to the UK Department for International Development. Building on his extensive experience in HIV, Global Health and International Development, Dr Conway has been building ixo (https://ixo.world), an ambitious project to create a blockchain-based Internet of Impact, which he describes as a ‘global digital immune system for humanity’. Dr Conway, expressing surprise at how quickly the years have flown by, sent his heartfelt congratulations to SAHCS and the SAJHIVMED for it’s milestone of 21 years.

Dr Conway’s tenure was followed by Prof. Linda-Gail Bekker (MBChB, DTMH, DCH, FCP[SA], PhD), who is Deputy Director of the Desmond Tutu Health Centre at the Institute of Infectious Disease and Molecular Medicine at the University of Cape Town (UCT) and Chief Operating Officer of the Desmond Tutu Health Foundation. Bekker is a physician-scientist and infectious disease specialist focusing on programmatic and action research around antiretroviral rollout, tuberculosis (TB) integration, and HIV prevention in key populations. She is Principal Investigator of the UCT Clinical Trials Unit funded by the US National Institutes of Health, and is actively involved in the work of its associated clinical research sites and networks. She has chaired protocols for the HIV Vaccine Trials Network and HIV Prevention Trials Network and has been the investigator of record in a number of network-related protocols. Prof. Bekker has served on numerous national, international and federal scientific and working committees, and is the current Chair of the SAHCS pre-exposure prophylaxis (PrEP) and post-exposure prophylaxis (PEP) guidelines. Bekker leads the Desmond Tutu Centre of Adolescent Health and Wellbeing at UCT, which aims to develop evidence-based best practices around adolescent treatment and prevention of HIV, TB and sexually transmitted infections (STIs), including the integration of these services within a robust, adolescent-friendly sexual and reproductive service platform. She was president of the International AIDS Society from 2016 to 2018.

Prof. Bekker, as SAJHIVMED Editor-in-Chief, wrote at the start of 2005 that:

Since inception of the journal in July 2000 an impressive list of guidelines has been compiled and peer reviewed by local experts. This year will also see the journal launched online, increasing accessibility of previous guidelines and journal articles.^2^

Prof. Bekker was the editor until late 2011 and has remained involved with the direction of the Journal by sitting on the editorial board of the SAJHIVMED since then. Prof. Bekker reminisced earlier this year that (personal communication, 2021):

‘The wonderful – and persuasive – Des Martin asked me to take this important role on when I was a relatively green-about-the ears HIV doctor and researcher. I was both thrilled and honoured and I loved the experience. My small role in the development of this important publication and its future wellbeing is something I am really proud to be counted a part of. I recall being particularly obsessed about getting the PubMed accreditation and understanding how that system worked. I’m delighted it has grown in both circulation and significance under the great curatorship of the recent editors.’

Prof. Landon Myer (MBBCH, PhD), who followed Prof. Bekker in the role of editor, is director and head of the School of Public Health and Family Medicine at the UCT. He had undergone training in social anthropology, clinical medicine and epidemiology. His research study focuses on women’s, maternal and child health in the context of HIV, which includes behavioural, clinical and health systems research. He has led multiple clinical and health systems studies investigating the health of HIV-infected women receiving antiretroviral therapy (ART) during pregnancy and postpartum, as well as the health and development of HIV-exposed and -infected children and adolescents. Within the School, he is professor and head of the Division of Epidemiology & Biostatistics, teaches epidemiological methods and is an academic convenor of the master’s in the public health programme. In Prof. Myer’s first editorial in 2011, he wrote that he was:

[*L*]ooking forward to continuing the journal’s emphasis on presenting research and clinical experiences from across the region, and keeping readers updated around local and international developments … by seeking to expand several sections, including: feedback reports from local and international conferences; editorial reviews intended to share viewpoints and promote discussion on important topics; and programmatic reports that share local experiences in implementing HIV treatment and prevention services on the ground.^3^

At the start of 2014, Prof. Myer whilst introducing a special anniversary edition, to mark 10 years of ART in the public sector, commented that:

In considering the HIV epidemic and its impact, many of our anniversaries are sad ones … Clinicians or scientists may mark the anniversary of the first documented AIDS case in a country, or the discovery of the virus itself, but these aren’t generally moments for celebration per se. So, it’s not often that we have cause to smile about an anniversary related to the epidemic. However, 1 April 2014 marks one happy anniversary worth remembering – a decade of antiretroviral therapy (ART) in the public sector. Like many anniversaries, the exact details can depend on where you were, and sometimes dates themselves can be fuzzy. Antiretrovirals were available from the 1990s in the private sector, and a trickle was accessible through trials and small donor-funded initiatives in urban centres from the early 2000s. Some provinces moved more quickly towards making ART available ahead of the National Department of Health, often with the assistance of partners in local and international non-governmental organisations. After the announcement of a national rollout of ART in public sector facilities, some hospitals received supplies of antiretrovirals within weeks. Elsewhere, especially in clinics in rural settings, health services took years to have local providers dispensing ART. Today the number of facilities dispensing ART is expanding still, but most communities across the country have reasonable access, and ART coverage continues to increase.^4^

Prof. Myer, reminiscing about his time as editor and the SAJHIVMED, said in 2021 (personal communication, Sept 9):

‘Another happy anniversary, worth pausing a moment to celebrate. The position of the SAHCS, and with it the journal, has shifted in the health landscape in South Africa – paralleling the shifting position of HIV – from addressing a single, specialised and focused health topic, to a cross-cutting, mainstream institution of sorts that is concerned with the health landscape of the country. It’s an incredible recognition of the central place of HIV in our health system and services – there is no concern that is more integral to our primary healthcare system than HIV and its associated conditions. It’s a credit in great part to the work of SAHCS. Congratulations to SAHCS and SAJHIVMED for continuing to provide important clinical updates, news and research, and being a leading voice of reason over the past 2 decades.’

Dr Michelle Moorhouse (MBBCh, DA, FRSPH) is a senior Global Medical Director at ViiV Healthcare. Prior to this, she worked at Ezintsha as Head of Treatment Strategies (2014–2019), where she was the co-Principal Investigator on the large Advance Study as well as working on other HIV treatment optimisation studies, policies and guidelines. Moorhouse was awarded an Honorary Clinical Fellowship at the Royal Free Hospital in London in 2003. Returning to South Africa in 2007, she re-established her general and HIV practice and set up a research centre focusing on HIV clinical trials, whilst consulting at an HIV clinic for an Eastern Cape based non-profit organisation (NPO). She served as a SAHCS board member between 2012 and 2018 and became a member of the Editorial Board of HIV Nursing Matters, shortly before becoming editor of the SAJHIVMED between 2015 and 2018. Her career has been focussed on making a difference in the lives of people affected by HIV (Moorhouse M, 2021, personal communication, Sept 9):

‘We always think of 21 as a “coming of age” or achieving maturity. I think the SAJHIVMED is an exception to this, in that right from the start it was clearly a powerful publication that has delivered high quality research, informative guidelines and great educational reads from which I personally have learned so much. A testament to the high calibre of the journal is the fact that it is listed on PubMed, which is a great achievement and one of which to be exceedingly proud. It was my great pleasure and privilege to be at the helm for a short while and I wish the journal continued growth over the next 21 years.’

Finally, in his first editorial mid-2019, Dr Dave Spencer (MBChB, MMed, DTM&H), current Editor-in-chief, summarised several key articles, so as to encourage journal readership, and stated that:

The articles address contemporary and regional issues in HIV medicine. The topics speak to all aspects of the epidemic: epidemiology, public health, prevention, clinical medicine, tuberculosis and opportunistic diseases, management guidelines, opinion pieces, editorials, and case reports. For the teachers, trainers, healthcare managers and administrators among us, there is a wealth of local information in these papers. Please acknowledge our talented researchers by reading what they write.^5^

Dr Spencer is a specialist physician who started treating HIV patients formally whilst completing a 2-year Infectious Diseases Fellowship at Case Western Reserve University in the United States, 1988–1990. In 1991, he took over as head of the HIV Clinic of the Johannesburg General Hospital. He was in private practice in Johannesburg from 1997 until 2011. Also trained in oncology and infectious diseases, Spencer was thereafter the Head of Infectious Diseases and ran the Themba Lethu HIV Clinic at Helen Joseph Hospital. Dr Spencer is renowned as a lecturer and teacher in the field of medicine that he loves. He is a sought-after speaker at continuing medical education (CME) meetings and other academic events. His book *The Clinical Practice of HIV Medicine* (2005) is a primer on HIV care for doctors and a summation of his experience as a practitioner in this field and is to be found on the desks of many practitioners. He has always been committed to imparting his knowledge through lectures, guidelines and one-on-one teaching in his clinic; he has taught HIV medicine throughout Africa, and many practitioners have benefitted from these sessions. Owing to his background and beliefs, he has been able to instil the need for assessing the patient more broadly than just paying attention to the physical components of the disease. Dr Spencer has published extensively in the field of HIV and was a local lead investigator for several early studies of ART. Finally, he was a founding executive member of SAHCS. Recently retired, Dr Spencer currently serves as the Editor-in-Chief of the SAJHIVMED, and is a key content developer, expert reviewer, teacher and guidelines contributor at SAHCS.

Dr. Spencer, who continues to identify key articles and summarise these in ‘newsletters’ to SAHCS’ members (2021, personal communication, September), often remarks:

‘The pages of the SAJHIVMED showcase many of the best of Africa’s researchers and clinicians. Original articles, opinion pieces, guidelines and reviews that emerge from the global epicentre of the HIV pandemic. What better place to understand the impact of an incurable disease on society and its people? What better place to do good and to influence the continent’s future?’

Southern African HIV Clinicians Society is proud to publish this anniversary special edition.

We express our sincerest gratitude to all the editors and peer reviewers who have contributed, and continue to contribute, to the growth of the journal. A special thanks to our authors whose impact on the SAJHIVMED continues to be felt. Thanks are also due to our publishers, AOSIS, who have walked the last few years of the SAJHIVMED journey together with SAHCS.


Prof Yunus Moosa Southern African HIV Clinicians Society President
Dr Lauren Jankelowitz Southern African HIV Clinicians Society Chief Executive Officer

